# Landscape of Apolipoprotein (APOE) in the Emirati Population and Recommendations for a Genotyping Approach for Amyloid-Beta Monoclonal Antibodies (Aβ-mAbs) in Alzheimer’s Disease Patients

**DOI:** 10.7759/cureus.113196

**Published:** 2026-07-22

**Authors:** Wadha Mohammed, Mohamed Al Ameri, Sahar Al Marzooqi, Habiba Alsafar, Roland Olbrich, Mira Mousa, Dalia Barayan, Tiago Magalhaes, Javier Quilez, Judith Reche, Budour Alkaf, Ahmed Shatila, Nour al dain Marzouka, Imran Iqbal, Ayman Yousif, Azhar Rahma, Asma Al Mannaie

**Affiliations:** 1 Genomics and Biobank, Department of Health - Abu Dhabi, Abu Dhabi, ARE; 2 Genetic and Molecular Biology, Khalifa University, Abu Dhabi, ARE; 3 Biomedical Engineering and Biotechnology, Khalifa University, Abu Dhabi, ARE; 4 Public Health and Epidemiology, Khalifa University, Abu Dhabi, ARE; 5 Bioinformatics, M42, Abu Dhabi, ARE; 6 Neurology Department, Sheikh Shakhbout Medical City, Abu Dhabi, ARE; 7 Genetics and Genomics, United Arab Emirates University, Abu Dhabi, ARE; 8 Specialist Trusted Research Environment, Department of Health - Abu Dhabi, Abu Dhabi, ARE; 9 Specialist Trusted Research Environment/Bioinformatics, Department of Health - Abu Dhabi, Abu Dhabi, ARE; 10 Public Health, United Arab Emirates University, Abu Dhabi, ARE; 11 Health Life Sciences, Department of Health - Abu Dhabi, Abu Dhabi, ARE

**Keywords:** alzheimer's disease, amyloid-related imaging abnormalities (aria), anti-amyloid-β monoclonal antibodies, apoe genotypes, emirati, emirati genome program, pharmacogenomic

## Abstract

Background: Apolipoprotein E (APOE) genotyping is increasingly recognized as a biomarker to support treatment decisions for Alzheimer’s disease (AD) with the introduction of anti-amyloid monoclonal antibodies as promising therapeutic options. Although not yet incorporated into formal pharmacogenomic guidelines, regulatory authorities acknowledge its potential role in advancing precision medicine approaches.

Methods: We reviewed AD cases diagnosed in 2024 from the Department of Health - Abu Dhabi records and assessed monoclonal antibody prescribing patterns. APOE genotypes were determined using the two defining variants, *APOE* rs429358 and rs7412, using whole-genome sequencing data from 506,734 participants enrolled in the Emirati Genome Program.

Results: In 2024, 1,285 individuals were diagnosed with AD. Aducanumab was prescribed to eight patients between 2022 and 2024. In the Emirati Genome Program cohort, the allele frequencies of rs429358 and rs7412 were 8.3% and 4.2%, respectively. The most prevalent APOE genotype was ε3/ε3 (77.9%). The APOE ε4 risk allele was observed in 14.7% of participants, including 0.9% who were homozygous for ε4, while the protective APOE ε2 allele was present in 7.5%.

Conclusion: APOE profiling supports genotype-informed treatment strategies for anti-amyloid therapies. These findings establish a population-specific framework for integrating genetic information into the Emirati Genome Program pharmacogenomic report, enhancing precision medicine approaches for AD management in the UAE.

## Introduction

Alzheimer’s disease (AD) is a neurodegenerative condition that primarily affects older adults, making it the most common cause of dementia [1]. Its earliest and most prominent symptom is often selective memory impairment, which may later progress to include language difficulties, disorientation, mood changes, behavioral disturbances, and impaired self-care abilities [2,3]. As research has advanced, several risk factors that increase the likelihood of developing AD have been identified, with age being the most significant risk factor. Additionally, sex, family history, and genetic predisposition play a role in AD risk [4-7].

AD typically manifests after age 65, a condition referred to as late-onset AD (LOAD) [4,5]. However, a subset of individuals may develop symptoms before this age, referred to as early-onset AD (EOAD), sometimes as early as the late forties [6]. With a rapidly aging global population and increasing life expectancy, the number of AD cases is rising. This number is projected to continue growing over the coming decades with minimal progress in treatment and prevention. In Arab countries, the burden of dementia appears to rise sharply with advancing age and given that AD is the leading cause of dementia globally, this trend is likely to be reflected in AD prevalence as well. A similar pattern has also been observed in Arab countries. For example, Qassem et al. (2023) reported that the prevalence of dementia among individuals aged 50 years and older in the United Arab Emirates (UAE) was 1.33%, increasing to 4.09% among those aged 60 years and older [8].

Monoclonal antibodies (mAbs) have recently emerged as a promising therapeutic approach for AD by selectively targeting key underlying pathogenic factors, such as amyloid-β (Aβ) plaques in the brain [9]. Based on their efficacy in slowing cognitive and functional decline in patients with AD, the U.S. Food and Drug Administration (FDA) has approved three anti-Aβ mAbs: aducanumab, lecanemab, and donanemab for clinical use [9]. The UAE was the second country globally to approve and register aducanumab for AD and has since approved both lecanemab and donanemab, expanding treatment options for selected individuals with AD [10]. Despite their therapeutic potential, the use of anti-Aβ mAbs raises significant safety concerns [11-13], particularly the occurrence of Amyloid-Related Imaging Abnormalities (ARIA), which manifest as vasogenic edema and/or sulcal effusions (ARIA-E) or brain superficial siderosis, microhemorrhages, and macrohemorrhages (ARIA-H) [14]. ARIA occurs more frequently in individuals with a genetic predisposition, with susceptibility influenced by specific allelic variants of the apolipoprotein E (APOE) gene.

The APOE gene is known to be a major cholesterol carrier that facilitates lipid transportation in the brain and plays a critical role in neural recovery [15]. The APOE gene is located on chromosome 19. It has three allelic isoforms: epsilon (ε) 2, 3, and 4 [15]. Since its identification in 1993, the (ε4) allele has been widely studied, with evidence confirming its association with cognitive decline and increased neuritic plaques, cerebral amyloid angiopathy, AD, and vascular dementia [15,16]. The risk associated with APOE ε4 varies by population, age, and sex, with risk being more pronounced in females than in males, and homozygosity for APOE ε4 representing the greatest risk [17,18]. Typically, having one ε4 allele raises AD risk by two- to threefold, while two copies increase the risk by eight- to twelvefold [19-21]. The APOE ε4 allele is also associated with the highest risk for adverse effects from new anti-Aβ mAbs, especially in homozygous carriers. Thus, determining the APOE genotype is recommended before treatment initiation to assess the risk of adverse outcomes. Considering these developments, this paper aims to provide insight into the utilization of anti-Aβ mAbs in the Emirate of Abu Dhabi, the characterization of APOE isoforms based on the Emirati Genome Program (EGP) dataset, and the integration of APOE ε4 genotyping into the EGP pharmacogenomic report.

## Materials and methods

Ethical considerations

Approval to conduct this study was obtained from the Abu Dhabi Health Research and Technology Committee (ADHRTC-2025-23) and the M42 Ethics Committee for access to and analysis of de-identified genetic and phenotypic data. The study complies with all applicable ethical regulations.

Patients identification and stratification

Patients with AD in Abu Dhabi were identified through the Department of Health (DoH) records. The cohort comprised all patients diagnosed using diagnostic codes from the 10th revision of the International Classification of Diseases (ICD-10), corresponding to the AD Phecodes G30.0, G30.1, and G30.9. Patients were further stratified by age (65 years and above versus younger than 65 years) and by gender. Additionally, utilization of anti Aβ-mAbs therapies was assessed using DoH-registered drug codes, including lecanemab (E37-C488-15317-01, E37-C488-15318-01, and E37-C488-17204-01), donanemab (E40-C480-15295-01), and aducanumab (K38-9738-10033-01).

ARIA reported cases

A dedicated pharmacovigilance division within the DoH monitors adverse drug reactions (ADRs). This division oversees reports submitted by healthcare facilities and takes appropriate action when ADRs are identified. Additionally, the division submits monthly reports to the Uppsala Monitoring Centre to support global signal detection efforts. Pharmacovigilance records were reviewed to identify reported ARIA cases.

Whole genome sequencing and quality control 

Through the EGP, 506,734 samples from Emirati participants were collected, with a mean age of 30.9 years (SD: 17.4). Whole genome sequencing was performed to a target depth of 30x on three platforms: Illumina (35.7%), MGI (62.4%), and Oxford Nanopore Technologies (ONT) (1.9%). Reads were aligned to the human genome assembly GRCh38 using DRAGEN Bio-IT Platform 3.9 (Illumina samples) [22], the BWA-MEM algorithm (MGI samples) [23], and Sentieon’s acceleration of Minimap2 (ONT samples) [24,25]. Variants were called using the DRAGEN Bio-IT Platform 3.9 (Illumina samples), Sentieon’s Haplotype Caller algorithm (MGI samples) [24], and Clair2 [26] and Sniffles2 [27] (ONT samples).

Genotype calls were subject to platform-specific quality thresholds (Illumina: GQ ≥10; MGI and ONT: DP ≥10), consistent with the EGP variant-level QC pipeline (Supplementary table 1 in Appendices). Individuals lacking a quality-passing genotype call at either SNP were excluded from isoform classification.

APOE genomic analysis

To identify the carrier status of the APOE isoforms, we conducted targeted genomic screening by extracting genotype data for the two well-established single-nucleotide polymorphisms (SNPs), rs7412 and rs429358, which define the APOE isoforms [28-30], from whole-genome variant call format (VCF) files using bcftools (v1.2) [31]. Allele frequencies were calculated within the Emirati population. Individuals heterozygous for the APOε4 allele were selected for further analysis. For phasing, we defined a genomic window spanning chr19:44780000-45034000, encompassing the APOE gene and its surrounding linkage disequilibrium region. Individual VCFs from APOε4 heterozygotes were merged using bcftools merge with the “-missing-to-ref” and “-force-samples” options to ensure consistent sample alignment and to fill missing genotypes as homozygous reference, accommodating the characteristics of whole-genome sequencing data. Phasing was then performed using Beagle (v5.4, 22Jul22.46e) [32] without an external reference panel. This approach ensured that phasing reflected the haplotype structure specific to the study population, avoiding potential bias introduced by population differences in publicly available reference panels. Individuals heterozygous at both rs429358 and rs7412 were assigned to the APOE ε2/ε4 diplotype based on phasing results.

Pharmacogenomic report

In collaboration between the DoH - Abu Dhabi and M42, the laboratory operator of the EGP, participants in the EGP are provided with pharmacogenomic reports to support the identification of genetic factors influencing drug metabolism, including APOE genotyping. M42 conducts DNA sequencing and automated report generation via a reporting engine incorporating tools for genotype identification, chromosomal phasing, and phenotype prediction, enabling individualized patient insights. The pharmacogenomics reports are generated from post-sequencing VCF files and other post-whole-genome sequencing (WGS) outputs, focusing on highly polymorphic pharmacogenes. These reports provide comprehensive insights into metabolizer types, their clinical implications, and guideline-based recommendations to help physicians tailor therapeutic plans based on each individual’s genetic makeup.

The report is generated by a fully automated, end-to-end auditable pipeline that includes a built-in seven-level quality control (QC) check to ensure result accuracy. The pipeline was designed to support population-level studies and is highly scalable, generating intuitive reports with actionable insights. The report recommendations align with standards from the Clinical Pharmacogenomics Implementation Consortium (CPIC), the Dutch Pharmacogenetics Working Group (DPWG), and the FDA to support personalized patient care. Physicians can access these reports through the Health Information Exchange platform (Malaffi), launched in 2019 as a key component of the digital transformation of the healthcare system in Abu Dhabi, which serves as a decision-support tool for more informed therapeutic planning. This integration allows physicians to easily access genetic insights within their clinical workflow, supporting personalized therapy.

## Results

Utilization of anti-Aβ mAb for AD in Abu Dhabi

**Figure 1:**
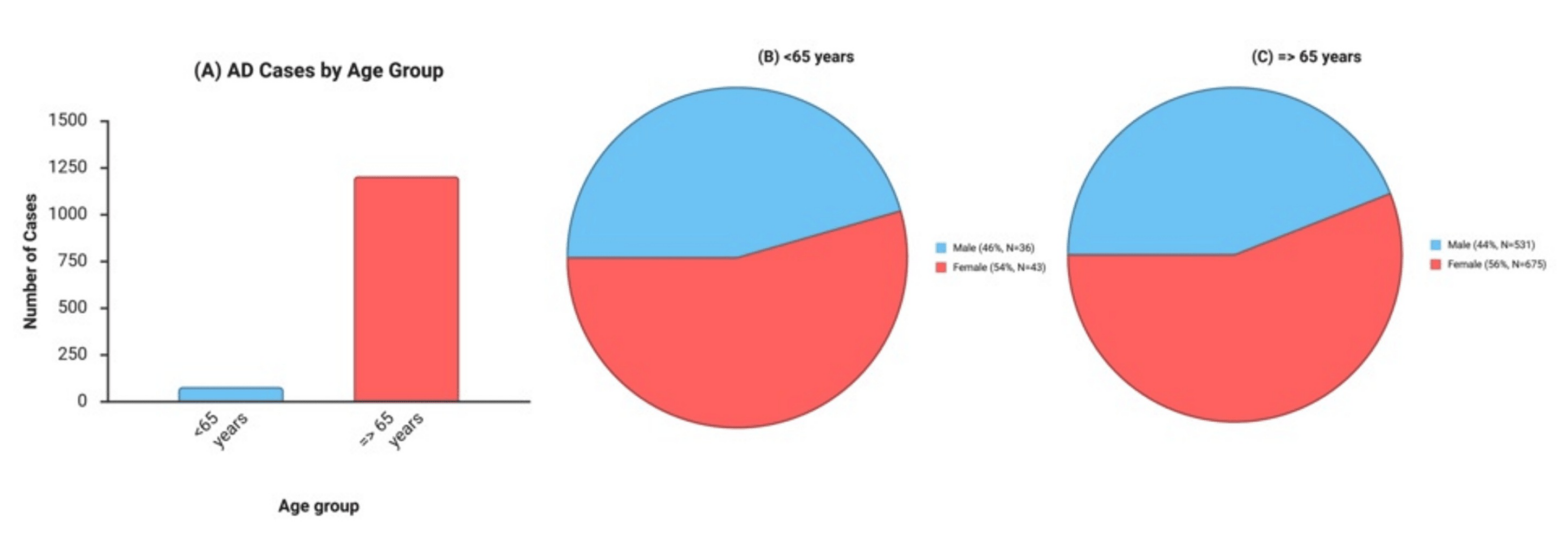
Alzheimer's disease cases in Abu Dhabi (n=1,285) Alzheimer's disease (AD) cases in Abu Dhabi by age and gender. AD cases among Emiratis in 2024 are shown by age group (A) and gender distribution within each group (<65 years in B, ≥65 years in C). Most cases occurred in individuals aged ≥65. Female cases slightly outnumbered male cases in both age groups.

Based on DoH records, 1,285 Emiratis were diagnosed with AD in Abu Dhabi in 2024 (Figure 1). Of these, 79 cases (6.1%) occurred in individuals under 65 years of age, comprising 43 females (3.3%) and 36 males (2.8%), while 1,206 cases (93.9%) occurred in individuals aged 65 and above, comprising 675 females (52.5%) and 531 males (41.3%). Among these patients, aducanumab was the only anti-Aβ mAb prescribed between 2022 and September 2024, as the manufacturer announced plans to discontinue the drug in 2024 to reprioritize resources toward AD research. It's important to note this decision was not attributed to safety or efficacy concerns [33]. According to DoH statistics and records, aducanumab remains registered and in use in Abu Dhabi at the time of this study, with a cumulative total of eight patients (0.6% of all AD patients) having received prescriptions during this period [34]. No patients received lecanemab or donanemab during the same timeframe. To date, no cases of ARIA-E or ARIA-H have been reported among AD patients receiving anti-Aβ mAbs in Abu Dhabi.

To support personalized and proactive clinical decision-making for AD patients, a pre-alert system has been implemented in selected healthcare facilities in Abu Dhabi. This system alerts physicians when anti-Aβ mAbs are prescribed, emphasizing the potential utility of APOE genotyping to optimize treatment plans, improve therapeutic outcomes, and individualize monitoring plans.

Distribution of APOE isoforms based on the EGP data

Population-specific allele frequencies (AFs) of two APOE SNPs (rs429358 and rs7412) were calculated using EGP cohort data (n = 506,734 Emiratis) and compared with global and regional frequencies from gnomAD (Table 1) [28,29,35,36]. The AF of rs429358 in the EGP cohort was 8.3%, which is lower than the overall gnomAD frequency (14.9%) and the non-Finnish European (NFE) subgroup (15.1%), but higher than the Middle Eastern (ME) subgroup (6.8%). Similarly, the AF of rs7412 in the EGP cohort was 4.2%, lower than both the overall gnomAD (7.4%) and NFE (7.8%) frequencies, and marginally below that of the ME subgroup (5.8%). Compared to the Qatari population (n = 216) in the National Center for Biotechnology Information (NCBI) dbSNP, which is geographically close to the UAE, the rs429358 variant was observed at a higher frequency, while rs7412 was found at a lower frequency than in the UAE cohort [35,36].

**Table 1:** Population-specific frequencies of APOE SNPs of interest ⁺ EGP allele frequencies with Wilson 95% confidence intervals: rs429358, 0.0830 (95% CI: 0.0830–0.0840); rs7412, 0.0420 (95% CI: 0.0420–0.0420). Confidence intervals are narrow owing to the large cohort size (n = 506,734 individuals; 1,013,468 alleles). SNP, single-nucleotide polymorphism; APOE, Apolipoprotein E; AF, allele frequency; EGP, Emirati Genome Program; GnomAD, Genome Aggregation Database; NFE, Non-Finnish European; ME, Middle Eastern; REF, reference allele; ALT, alternate allele.

SNP	CHR	Position (GRCh38)	REF	ALT	EGP⁺	dbSNP Qatar	GnomAD Overall	GnomAD NFE	GnomAD ME
rs429358	chr19	44,908,684	T	C	0.083	0.106	0.149	0.151	0.068
rs7412	chr19	44,908,822	C	T	0.042	0.014	0.074	0.078	0.058

In accordance with established findings in other populations [37-39], the most prevalent APOE isoform in the Emirati population was APOE ε3, observed in 77.9% of individuals as the APOE ε3/ε3 genotype. Conversely, the rarest isoform globally [40], APOE ε1, was also the least common in this cohort, with no individuals identified as homozygous (APOE ε1/ε1) and only 7 identified across all heterozygous ε1 genotypes (APOE ε1/ε4, n = 5, 0.0010%; APOE ε1/ε2, n =2, 0.0004%). The near-absence of the ε1 haplotype in this cohort further supports the classification of all double heterozygous individuals (at both rs429358 and rs7412) as APOE ε2/ε4 rather than ε1/ε3, since the ε1/ε3 diplotype has been previously reported as an incidental finding in isolated families worldwide [41]. The protective APOE ε2 isoform was present in 7.5% of the population, including 0.3% homozygous (APOE ε2/ε2) and 7.2% heterozygous (APOE ε2/ε3) individuals. The APOE ε4 isoform, which is associated with the highest risk for LOAD, was observed in 14.8% of the EGP cohort. This included 0.9% homozygous carriers (APOE ε4/ε4) and 13.9% heterozygous individuals (APOE ε1/ε4, APOE ε2/ε4, and APOE ε3/ε4) (Table 2).

**Table 2:** Prevalence of APOE isoforms in the EGP (n=506,734) 95% CI: Wilson 95% confidence intervals computed for each genotype proportion (n = 506,734). ⁺ APOE ε2/ε4 and APOE ε1/ε3 produce identical unphased genotypes at rs429358 and rs7412. Haplotype phasing resolved individuals in this category to ε2/ε4, consistent with the near-absence of the ε1 haplotype (ε1/ε1 = 0; ε1/ε2 = 2; ε1/ε4 = 5) and with Hardy–Weinberg expectation based on the observed ε2 (4.2%) and ε4 (8.3%) allele frequencies. APOE, Apolipoprotein E; EGP, Emirati Genome Program; CI, Confidence interval.

Isoform	rs429358	rs7412	Individuals (n)	Prevalence (%)	95% CI	Males (%)	Females (%)	Comment
					(%)			
APOE ε4 Isoforms								
APOE ε1/ε4	(C;C)	(C;T)	5	0.00%	0.0004–0.0023%	20	80	
APOE ε2/ε4⁺	(C;T)	(C;T)	3,108	0.61%	0.5920–0.6350%	41	59	
APOE ε3/ε4	(C;T)	(C;C)	66,730	13.17%	13.0760–13.2620%	42.2	57.8	
APOE ε4/ε4	(C;C)	(C;C)	4,349	0.86%	0.8330–0.8840%	41.4	58.6	~11× increased Alzheimer’s risk
Other Isoforms								
APOE ε1/ε1	(C;C)	(T;T)	0	-	-	-	-	Rare missing allele
APOE ε1/ε2	(C;T)	(T;T)	2	0.00%	0.0001–0.0014%	50	50	
APOE ε2/ε2	(T;T)	(T;T)	1,372	0.27%	0.2570–0.2850%	43	57	Protective isoform - Lowest risk
APOE ε2/ε3	(T;T)	(C;T)	36,053	7.12%	7.0440–7.1860%	42.1	57.9	
APOE ε3/ε3	(T;T)	(C;C)	395,115	77.97%	77.8590–78.0870%	42.5	57.5	Most common - Does not influence risk

Age and sex-stratified distributions of APOE ε4 heterozygotes and homozygotes are presented in Supplementary table 2 (Appendices). APOE ε4 heterozygote prevalence was consistent across all age groups, ranging from 14.0% in individuals aged >70 years to 16.8% in those aged 0-18 years, while APOE ε4/ε4 homozygote prevalence ranged from 0.8% to 1.1% across the same strata. Notably, carrier frequencies in the >50-70 and >70 age groups were comparable to those observed in younger strata, indicating that population-level estimates are consistent across the age range relevant to anti-Aβ mAbs therapy candidacy. A modest reduction in ε4 carrier frequency was observed in the >70 group relative to middle-aged strata, which may be related to the known association between the ε4 allele and increased cardiovascular and neurological mortality [42].

## Discussion

As the treatment landscape for AD continues to evolve, integrating genetic insights into clinical decision-making is becoming increasingly important. While the small number of treated individuals (n = 8) limits conclusions regarding the safety profile of anti-Aβ-mAbs in the Emirati population, the genetic data presented here provide an important foundation for future clinical planning and policy development. This study provides a population-specific characterization of APOE allele and genotype frequencies in the Emirati population, establishing a genomic reference to support the proactive implementation of APOE genotyping within the EGP pharmacogenomic report and to inform future clinical decision-making and healthcare planning. APOE allele and genotype frequencies observed in the EGP cohort may not directly reflect those of older individuals with AD or those who are eligible for anti-Aβ mAb therapies, as the analysis was conducted in a large population-based Emirati cohort rather than in a disease-specific population.

In this context, our findings offer timely and actionable insights. The determined APOE ε4/ε4 allele and genotype frequencies in the Emirati population have direct clinical and economic implications for the introduction and use of anti-Aβ mAbs in the UAE. These frequencies inform the proportion of EGP participants who would be classified as being at high or moderate risk for ARIA, thereby influencing future treatment selection, anticipated healthcare burden, and the need for increased MRI monitoring. By incorporating pharmacogenomic data into clinical workflows, UAE clinicians can align with global precision medicine trends while addressing region-specific needs.

Comparison with the ME population in gnomAD and the Qatari population in NCBI dbSNP revealed notable differences in variant frequencies. Some variation is expected, as gnomAD represents a broader regional cohort, whereas the Qatari data reflect a single, geographically close population. However, the small sample sizes in both databases (gnomAD ME and Qatari) may also contribute to bias [28,29,35,36]. In contrast, our large population-based cohort (>500,000 individuals) offers a more reliable and representative view, underscoring the need for population-specific genomic databases to support accurate clinical interpretation.

International regulatory practices for integration of APOE genotyping within pharmacogenomics reports remain inconsistent. APOE testing is not currently included in CPIC or DPWG guidelines. The United Kingdom’s Medicines and Healthcare Products Regulatory Agency excludes APOE ε4 homozygotes from receiving lecanemab, while the European Medicines Agency recommends restricting lecanemab to patients with no more than one copy of the ε4 allele [43]. The FDA recommends that APOE genotyping be performed before initiating treatment with aducanumab or lecanemab, a recommendation also reflected in the Pharmacogenomics Knowledgebase (PharmGKB) [44]. These regulatory discrepancies reflect a lack of harmonization in global policy and underscore the need for context-specific, evidence-based guidelines tailored to national populations.

By leveraging large-scale genomic data and implementing pharmacogenomic reporting, we aim to support clinicians and patients in making informed, evidence-based decisions that balance therapeutic benefits with potential risks. This approach aligns with global trends in precision medicine and addresses the specific needs and genetic landscape of the UAE population. Given these implications, we recommend routine APOE genotyping for Emirati patients being considered for anti-Aβ mAb therapies, supported by comprehensive clinical pathways that include culturally sensitive counseling for patients and their families. Counseling should address individualized ARIA risks, the benefits and limitations of therapy, monitoring needs, and related logistical and financial considerations. Ethical challenges, such as risk misinterpretation, genetic discrimination, and potential psychological effects on patients and their families, must be managed through robust education strategies, privacy safeguards, and access to certified genetic counselors.

## Conclusions

In conclusion, although the number of patients currently receiving anti-Aβ mAbs therapy in 2024 remains low, it is expected to rise given the increasing prevalence of dementia and AD both globally and regionally, alongside the growing availability of disease-modifying therapies. In this context, a pre-emptive, population-scale pharmacogenomics reporting approach supported by a clinical decision support tool offers a proactive strategy to optimize outcomes as eligibility for these therapies expands. Incorporating APOE genotyping into the pharmacogenomics report is particularly cost-effective, as APOE status is a critical determinant of ARIA risk; identifying at-risk patients before treatment initiation can reduce the clinical and economic burden associated with ARIA-related monitoring, management, and adverse outcomes. These findings are therefore not only scientifically novel, but also have clinical implications. They support the development of national clinical guidelines for APOE genotyping and anti-Aβ mAb administration in the UAE. Such guidelines should be grounded in local genetic data, aligned with international best practices, and supported by existing pharmacogenomic infrastructure, ensuring personalized, ethical, and culturally appropriate care.

## Appendices

**Table 3:** Supplementary table 1: Summary of quality control thresholds applied at both sample and variant levels across different sequencing platforms WGS, whole-genome sequencing; GQ, genotype quality; DP, read depth; SNP, single-nucleotide polymorphism; QUAL, variant quality score; ONT, Oxford Nanopore Technologies. Sample-level QC ensures sufficient depth, low contamination, and reliable genotype data. Variant-level QC thresholds for genotype quality (GQ) and read depth (DP) are platform- and caller-specific.

Parameter	Threshold / Criteria	Notes		
Sample-Level Quality Control				
Mean effective genome coverage	≥26x	Ensures sufficient depth for reliable variant calling		
% of genome with ≥10x coverage	≥95%	Measures uniformity of coverage		
Percentage of >Q30 bases	≥85%	Reflects base-level quality		
Yield / Total number of bases	≥90 Gb	Sufficient raw data generation		
Mapping Rate	≥70%	Proportion of reads that align to the reference genome		
Genotype missingness	<5%	Individuals with >5% missing genotypes are excluded		
Het/Hom Ratio	≤2.5	High ratios may indicate contamination or sample issues		
Contamination	≤1%	As estimated from WGS contamination detection tools		
Sex Concordance	Matches reported sex	Based on sex chromosome genotype consistency		
Variant-Level Quality Control				
Platform	Caller	Filters used	Genotype Quality (GQ)	Depth (DP)
Illumina	DRAGEN	Yes (variant flags)	SNP GQ ≥10.41; Indel GQ ≥7.83	DP ≥1
MGI	Sentieon / ZBOLT	No flags used	GATK HaplotypeCaller; GQ ≥30 recommended	DP ≥10
ONT	Clair3	Yes (variant flags)	QUAL filters between 2–5	DP ≥10

**Table 4:** Supplementary table 2: Age- and sex-stratified prevalence of APOE ε4 carriers in the EGP (n = 504,000 screened) Of 69,843 APOE ε4 heterozygotes and 4,349 APOE ε4/ε4 homozygotes identified in the full cohort (Table 2), age data were available for 69,833 (>99.9%) heterozygotes and 4,349 (100%) homozygotes; both age and sex (male/female) data were available for 68,955 (99.9%) heterozygotes and 4,287 (98.6%) homozygotes, who are included in the age- and sex-stratified rows below. Percentages represent APOE ε4 carrier prevalence within each age group relative to the total number of individuals with complete age and sex data in that stratum (Overall: n = 450,816; Males: n = 214,119; Females: n = 236,303). † APOE ε4 heterozygotes include APOE ε1/ε4, ε2/ε4, and APOE ε3/ε4 (the ε1/ε4 category comprised 5 individuals). APOE, Apolipoprotein E; EGP, Emirati Genome Program; SD, standard deviation.

Age Group	APOE ε4 Heterozygotes† n (%)	APOE ε4/ε4 Homozygotes n (%)
Overall		
0–18	19,787 16.79%)	1,247 (1.058%)
>18–35	24,598 (14.80%)	1,516 (0.912%)
>35–50	15,417 (14.70%)	930 (0.887%)
>50–70	7,658 (14.97%)	511 (0.999%)
>70	1,495 (14.02%)	83 (0.778%)
Total	68,955	4,287
Mean Age, years (SD)	29.9 (17.6)	30.1 (17.6)
Males		
0–18	9,521 (16.94%)	582 (1.036%)
>18–35	12,290 (14.53%)	738 (0.872%)
>35–50	6,802 (14.28%)	415 (0.871%)
>50–70	2,984 (14.26%)	194 (0.927%)
>70	662 (13.96%)	42 (0.886%)
Total	32,259	1,971
Mean Age, years (SD)	28.7 (17.2)	29.0 (17.4)
Females		
0–18	10,266 (16.67%)	665 (1.080%)
>18–35	12,308 (15.10%)	778 (0.955%)
>35–50	8,615 (15.07%)	515 (0.901%)
>50–70	4,674 (15.48%)	317 (1.050%)
>70	833 (14.15%)	41 (0.696%)
Total	36,696	2,316
Mean Age, years (SD)	31.1 (17.8)	31.0 (17.8)
